# RNAi targeting Nogo Receptor enhanced survival and proliferation of murine retinal ganglion cells during N-methyl-D-aspartate-induced optic nerve crush

**DOI:** 10.18632/oncotarget.17351

**Published:** 2017-04-21

**Authors:** Kun Zeng, Bo Zhong, Xiao-Li Shen, Min Fang, Bao-Tao Lin, Da-Hui Ma

**Affiliations:** ^1^ Key Laboratory of Ophthalmology, Shenzhen Eye Hospital, Ophthalmology College of Shenzhen University, Shenzhen 518000, P.R. China; ^2^ Department of Stomatology, Shenzhen Second People’s Hospital, Shenzhen 518000, P.R. China

**Keywords:** Nogo receptor, RNA interference, proliferation, apoptosis, mouse retinal ganglion cells

## Abstract

We investigated the effects of lentivirus-mediated RNAi targeting of Nogo Receptor (*NgR*) on the proliferation and survival of murine retinal ganglion cells (mRGCs) *in vitro* and *in vivo*. Cultured mRGCs and C57BL/6 male mice were divided into 4 experimental groups: blank, model [100 μM N-methyl-D-aspartate (NMDA)], nscRNA (100 μM NMDA+ nscRNA vectors) and siNgR (100 μM NMDA+ siNgR vectors). CCK-8 and flow cytometry analyses revealed that silencing *NgR* enhanced proliferation, cell cycling and survival of NMDA-treated mRGCs. H&E staining showed that *NgR* silencing enhanced mRGC cell density and reduced angiogenesis in NMDA-treated retinal tissues. TUNEL assays showed that mRGC apoptosis was significantly diminished by *NgR* silencing in NMDA-treated retinal tissues. Western blotting and qRT-PCR analysis in NMDA-treated mRGCs and murine retinal tissues revealed that *NgR* silencing resulted in downregulation of RhoA signaling (RhoA and ROCK2). Western blotting showed that levels of activated Bax and cleaved caspase 3 were decreased, while Bcl-2 and pro-caspase 3 were increased in NMDA-treated mRGCs and murine retinal tissues, which corroborated the decreased apoptosis. These findings indicate that *NgR* gene silencing increases proliferation and survival of mRGCs in NMDA-treated murine retinas, which suggests a potential for therapeutic application to preventing optic nerve damage.

## INTRODUCTION

Retinal ganglion cells (RGCs) play a critical role in integrating visual signals and processing within the eyes [1]. The axons of the RGCs form the optic nerve, which constantly transmits messages from the retina to the brain [2]. When the axons are injured, RGCs undergo programmed cell death or apoptosis resulting in irreversible loss of function [3]. Apoptosis is a systematic program of cell death that occurs in normal physiological or pathological conditions when cells have been damaged beyond repair [4]. Selective RGC apoptosis occurs not only during traumatic optic nerve injury, but has also been reported in the retinal pathology of various optic neuropathies [5]. The previous study has explored neuroprotective therapies to reduce or decelerate axotomy-induced apoptosis of RGCs [3]. However, the complex pathophysiology of RGC death still remains unclear [6]. Hence, identifying the key players that regulate the apoptotic pathways of the retinal cells is necessary in order to develop therapeutic inhibitors that can enhance RGC survival and protect the optic nerve. Although numerous genes that differentially regulate RGC death have been identified in recent studies [7–9], further investigations into the detailed mechanism of RGC apoptosis is of paramount importance

Nogo receptors (*NgRs*), also known as reticulon 4 receptors (RTN4R) or Nogo-66 receptors are members of the glycosylphosphatidylinositol-anchored family of membrane-bound cell surface receptors [10]. These receptors are highly expressed by neurons in regions with high plasticity that also demonstrates elevated Nogo expression [11]. In mammalian brains, *NgRs* are densely distributed in the axonal, dendritic and spine membranes and in both pre- and post-synaptic density fractions [10]. *NgRs* are also highly expressed in the RGCs of retina [12]. Besides, *NgRs* are associated with many diseases including a variety of neurological disorders and multiple sclerosis [13]. They are regarded as components of a signaling axis that inhibits neuronal regeneration after central nervous system injury [13]. Activated *NgRs* can suppress nerve regeneration, which results in neuron atrophy and apoptosis [14]. In recent years, RNA interference (RNAi) has been extensively used to study regulation of gene expression and therapeutic intervention for many diseases, including cancers [15]. Also, a variety of knockout or transgenic mice are available that are amenable for RNAi technology in order to investigate the *in vivo* roles of various proteins [16]. In this study, we used lentivirus-mediated RNAi of *NgR* and explored its effects on the proliferation and survival of mRGCs and optic nerve protection.

## RESULTS

### RNAi of NgR promoted proliferation of NMDA-treated mRGCs

**Figure 1:**
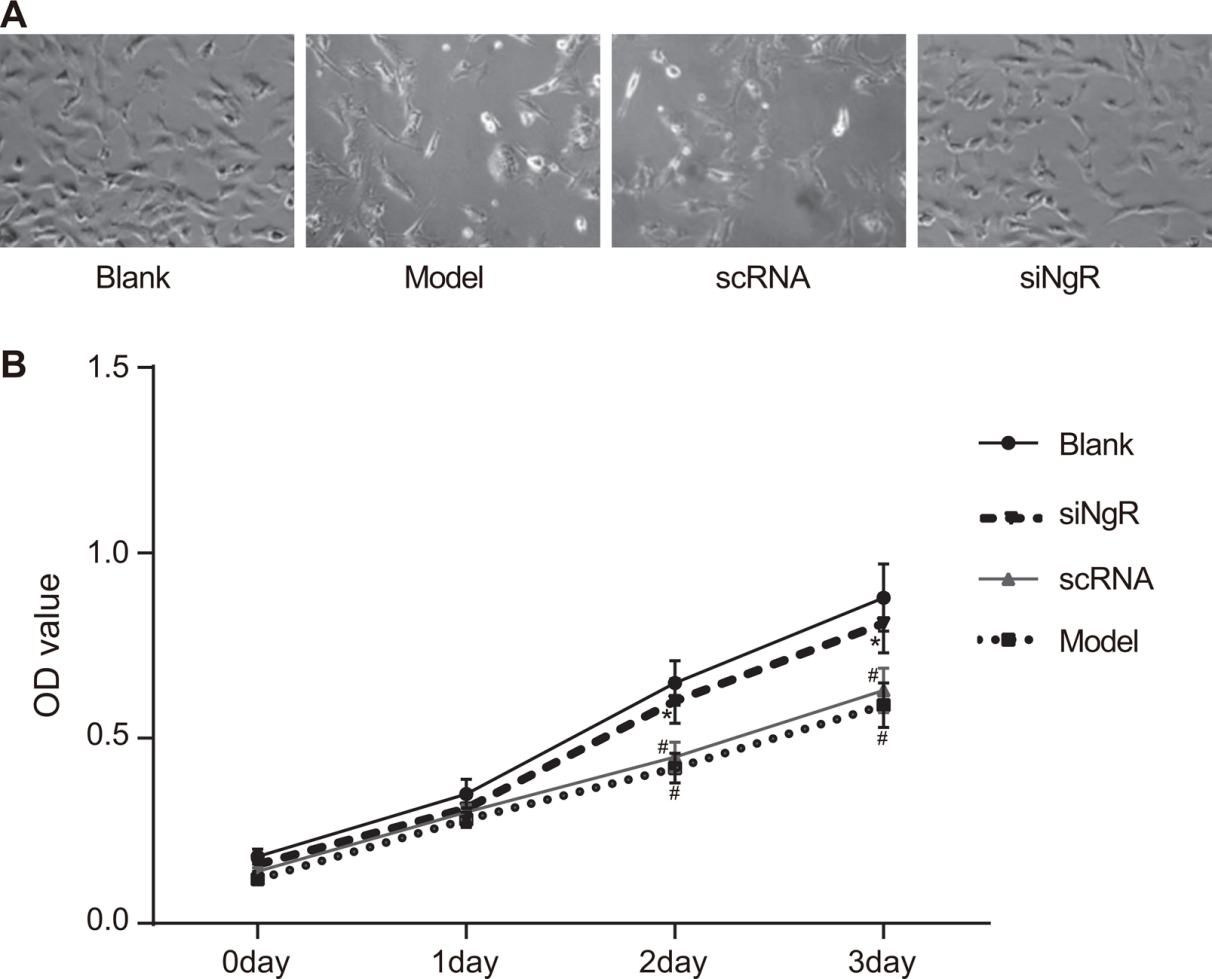
*NgR* gene silencing promotes proliferation of NMDA-treated mRGCs (**A**) The total number of mRGCs were quantified by counting under a light microscope in the 4 experimental groups of mRGCs that were cultured for 3 days. (**B**) The proliferation of mRGCs from the 4 experimental groups was quantified on days 0–3 by CCK-8 assay. Optical density measurement was performed at 490 nm. ^#^*P* < 0.05 compared to the blank group; *P < 0.05 compared to the model and scRNA groups; mRGCs, mouse retinal ganglion cells.

We analyzed the effects of *NgR* knockdown on the proliferation of cultured mRGCs treated with *N-*methyl-D-aspartate (NMDA), which induced mRGC apoptosis by cell count and CCK8 assays. We observed that compared to the blank group, mRGCs in the model group showed significant reduction of growth demonstrating the effect of NMDA treatment (*P* < 0.05; Figure 1A–1B). Further, we observed that proliferation of mRGCs in the model and the nscRNA groups was significantly lower compared to the blank group (*P* < 0.05) and the siNgR group (*P* < 0.001) suggesting that silencing of *NgR* enhanced proliferation of mRGCs (Figure 1A–1B). We also observed that all 4 groups of mRGCs showed similar proliferation rates on days 0 and 1 (*P* > 0.05). No significant differences were observed between the nscRNA and the model groups (all *P* > 0.05) as well as the siNgR and the blank groups (all *P* > 0.05) (Figure 1A–1B). These data suggested that silencing *NgR* resulted in enhanced proliferation of NMDA-treated mRGCs.

### NgR silencing promoted cell cycle progression of NMDA-treated mRGCs

**Figure 2:**
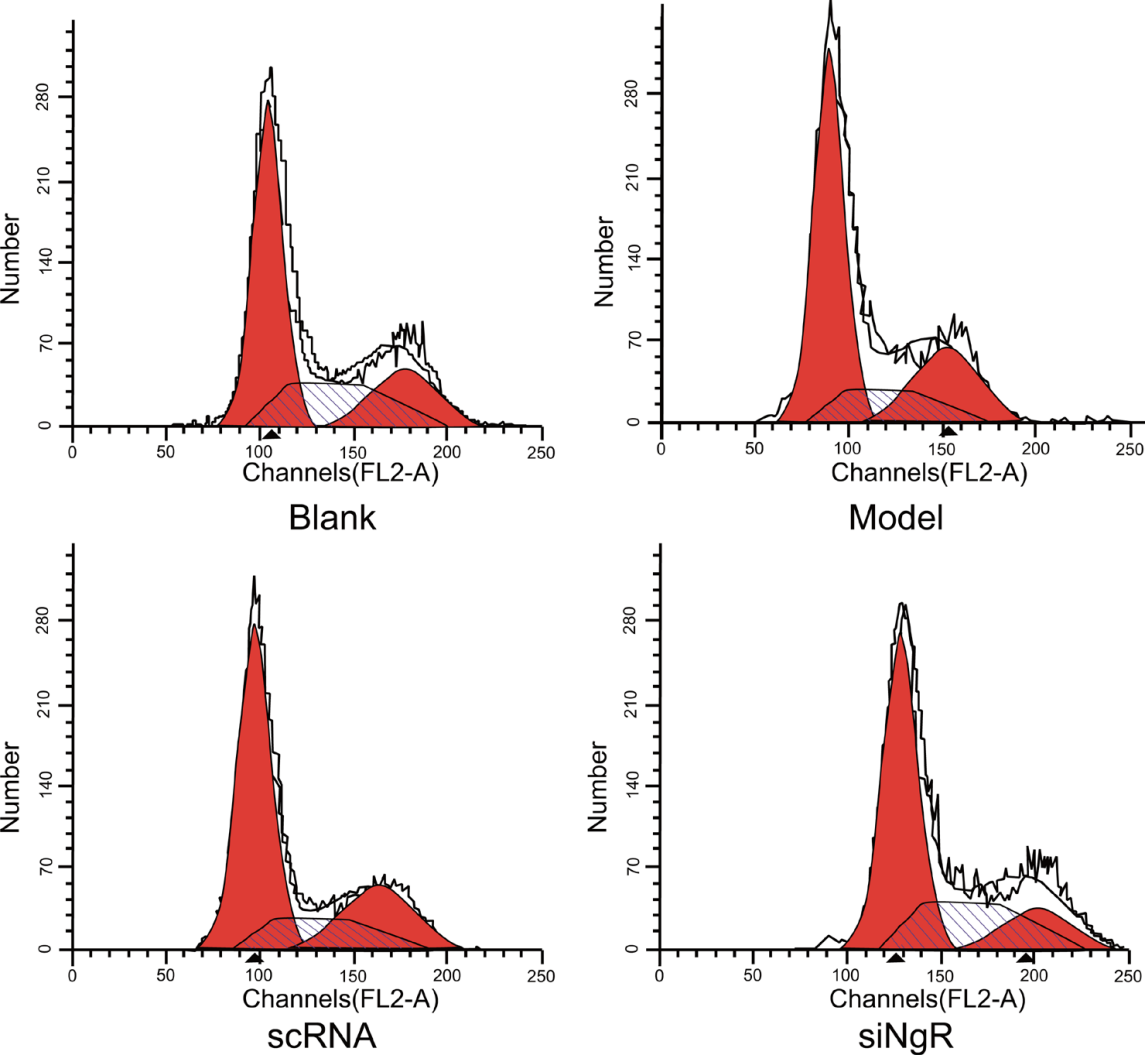
Effects of *NgR* gene silencing on cell cycle progression of NMDA-treated mRGCs Propidium iodide stained mRGCs from the 4 experimental groups that were grown for 3 days were analyzed by flow cytometry. Note: ^#^*P* < 0.001 compared to the blank group; *P < 0.001 compared to the model and scRNA groups; mRGCs, mouse retinal ganglion cells.

Next, we analyzed the effect of silencing *NgR* on cell cycle parameters of mRGCs using PI staining and flow cytometry. We observed significant decrease in percentage of S-phase cells (*P* < 0.001; Figure 2) in the model and nscRNA groups and significant increase in the percentage of G2/M-phase cells in comparison with the blank and siNgR groups (*P* < 0.001 for blank; *P* < 0.05 for siNgR; Figure 2). However, the percentage of G0/G1-phase cells was comparable among all 4 groups (*P* > 0.05; Figure 2). Both the nscRNA and model groups showed similar percentage of G0/G1, S and G2/M phase cells (*P* > 0.05; Figure 2, Table 1); and both the siNgR and blank groups had comparatively similar percentage of G0/G1, S and G2/M phase cells (*P* > 0.05; Figure 2, Table 1). These results suggested that *NgR* knockdown increased DNA replication and cell cycle progression in NMDA-treated mRGCs.

**Table 1:** Effects of NgR gene silencing on cell cycle of mRGCs detected by flow cytometry

Group	G0/G1	S	G2/M
Blank	70.23 ± 3.68	16.72 ± 1.49*	13.05 ± 4.77*
Model	68.67 ± 2.28	8.23 ± 1.34^#^	23.10 ± 2.75
scRNA	68.34 ± 4.32	7.82 ± 0.62^#^	23.84 ± 4.91
siNgR	70.76 ± 3.75	17.21 ± 1.89*	12.03 ± 4.27*

Note: ^#^*P* < 0.001 compared with the blank group; *P < 0.001 compared with the model and scRNA groups; RNAi, RNA interference; mRGCs, mouse retinal ganglion cells.

### NgR silencing reduced apoptosis of NMDA-treated mRGCs

**Figure 3:**
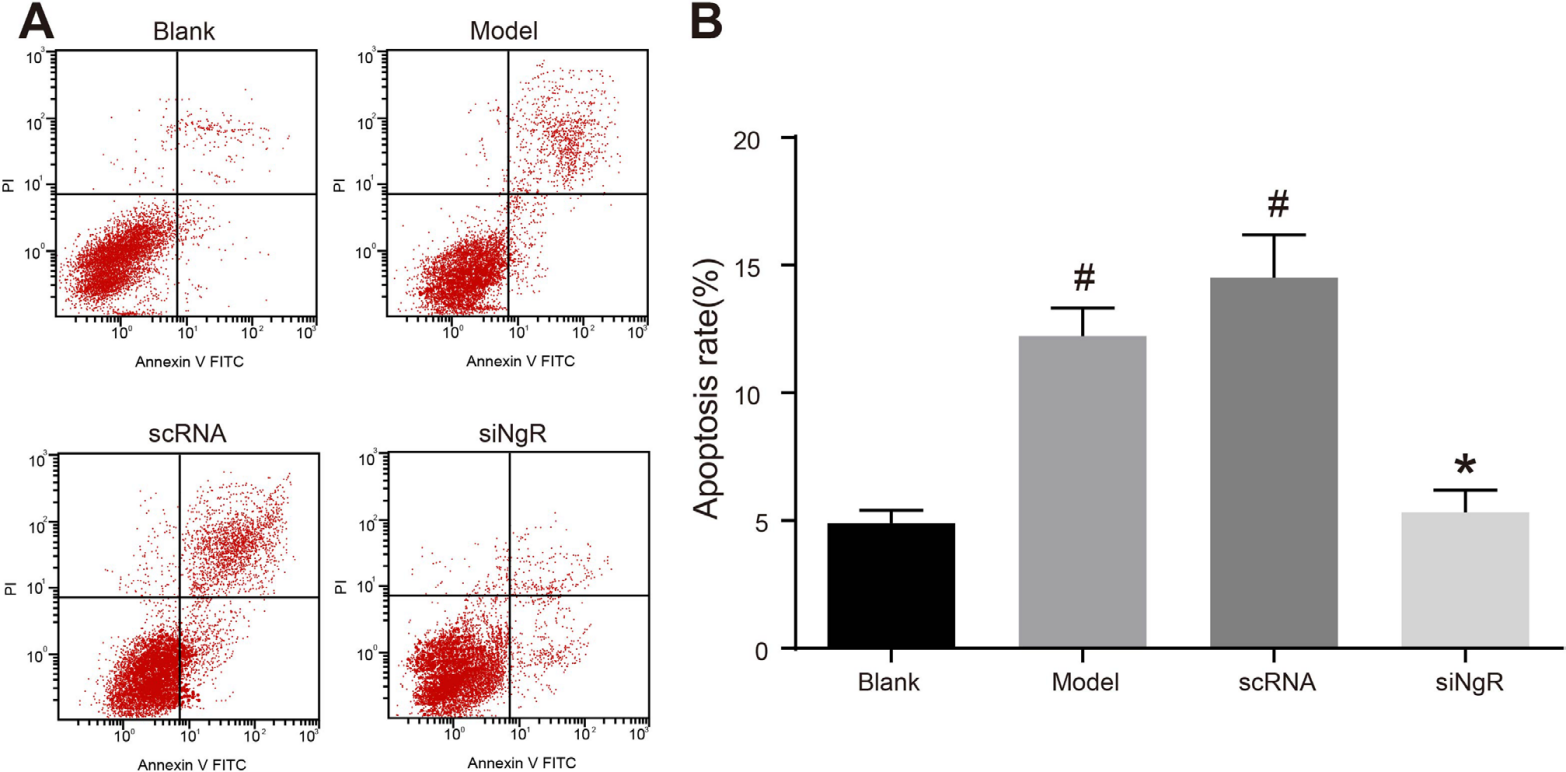
*NgR* gene silencing significantly inhibits apoptosis of NMDA-treated mRGCs (**A**) Flow cytometry analysis of the percent apoptosis in the 4 groups of mRGCs cultured for 3 days was determined by AnnexinV/PI double staining. AnnexinV^+^/PI^+^ mRGCs are considered apoptotic. (**B**) Apoptotic rate was analyzed in each of the 4 groups of mRGCs. ^#^*P* < 0.001 as compared with the blank group; *P < 0.001 as compared with the model and scRNA groups; mRGCs, mouse retinal ganglion cells.

Further, we analyzed the effects of *NgR* silencing on apoptosis of NMDA-treated mRGCs using flow cytometry analysis of AnnexinV-PI double stained mRGCs from the 4 experimental treatment groups. We observed that the apoptotic rates (AnnexinV^+^ and PI^+^) in the model and the nscRNA groups were significantly higher compared to the blank and siNgR groups (*P* < 0.001; Figure 3). The apoptotic rates in the siNgR and blank groups were comparable (*P* > 0.05; Figure 3). This suggested that knockdown of *NgR* reduced apoptosis in the NMDA-treated mRGCs.

### NgR silencing decreased RhoA signaling in NMDA-treated mRGCs

**Figure 4:**
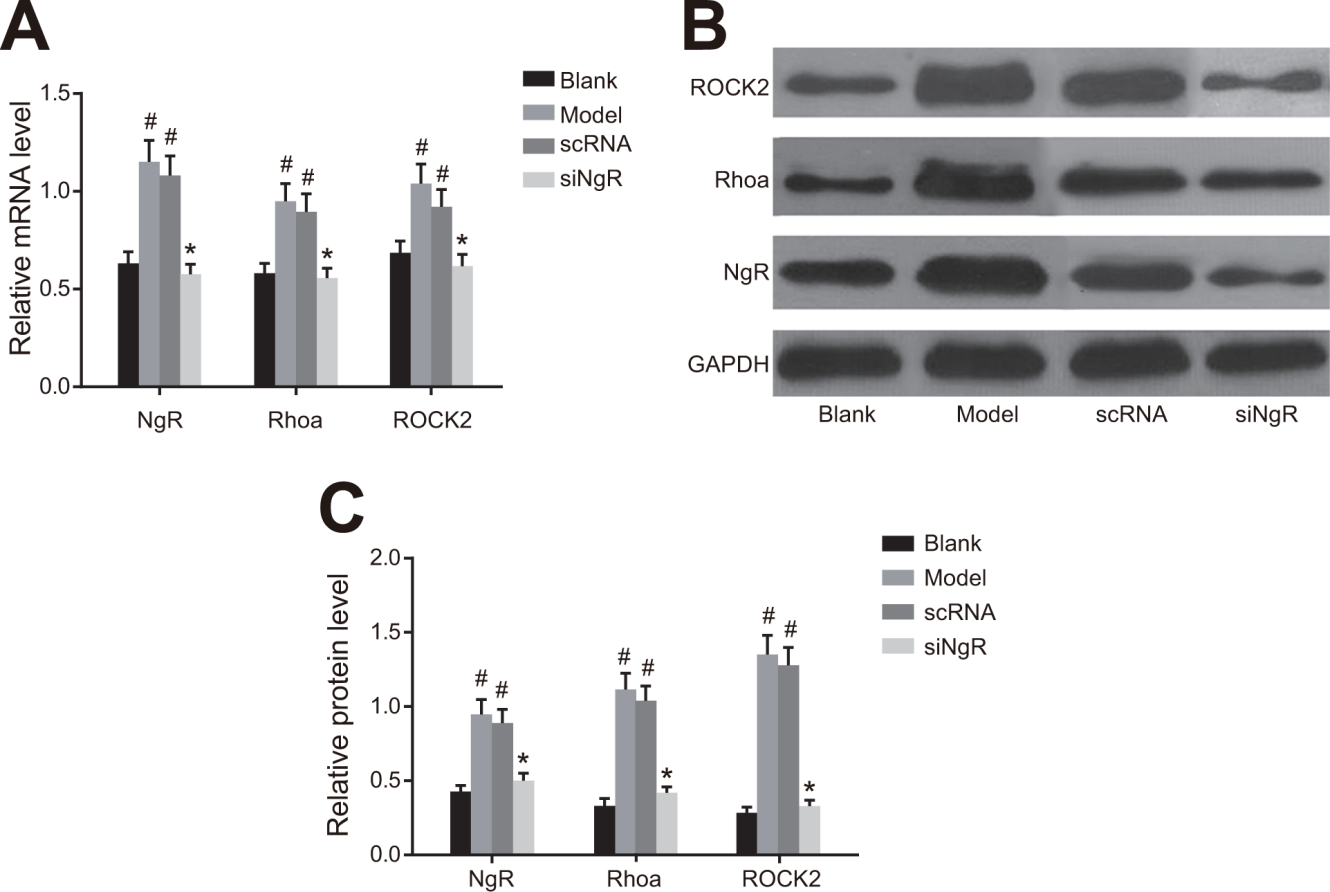
The effect of *NgR* silencing on RhoA signaling pathway in NMDA-treated mRGCs (**A**) The expression of *NgR*, RhoA and ROCK2 mRNAs was determined by qRT-PCR in the 4 groups of mRGCs cultured for 3 days. (**B**) Western blotting analysis of protein expression of *NgR*, RhoA and ROCK2 in the 4 groups of mRGCs cultured for 3 days. (**C**) The gray values for *NgR*, RhoA and ROCK2 proteins in the 4 groups of mRGCs is shown. ^#^*P* < 0.001 as compared with the blank group; *P < 0.001 as compared with the model and nscRNA groups; mRGC, mouse retinal ganglion cells; qRT-PCR, quantitative real-time polymerase chain reaction.

We analyzed the status of the RhoA signaling pathway that is downstream of *NgR* in NMDA-treated mRGCs by qRT-PCR and western blotting analysis of *NgR*, RhoA and ROCK2 in the 4 groups of mRGCs (Figure 4). We observed that expression of *NgR*, RhoA and ROCK2 mRNAs and proteins were significantly higher in the model and the nscRNA groups compared with the blank and siNgR groups (*P* < 0.001; Figure 4). This suggested that *NgR* silencing downregulated the RhoA signaling pathway in NMDA-treated mRGCs.

### NgR silencing increased Bcl-2 and pro-caspase-3 and decreased activated Bax and cleaved caspase-3 in NMDA-treated mRGCs

**Figure 5:**
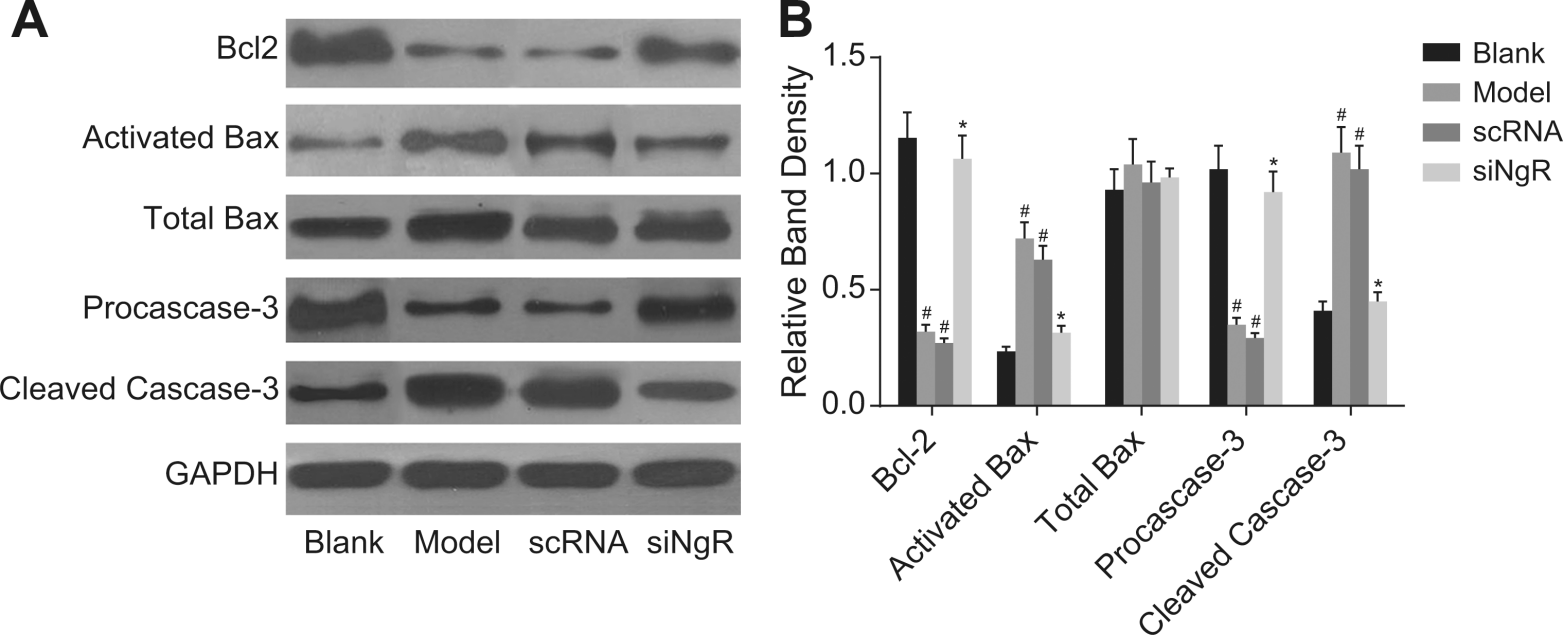
The effect of *NgR* silencing on expression of apoptosis-related proteins in NMDA-treated mRGCs (**A**) Western blotting analysis of Bcl-2, activated Bax, total Bax, pro-caspase3 and cleaved caspase3 in the 4 mRGC groups after culturing for 3 days. (**B**) The relative expression of the apoptosis-related proteins (Bcl-2, activated Bax, total Bax, pro-caspase3 and cleaved caspase3) normalized to GAPDH expression is shown. ^#^*P* < 0.001 compared with the blank group; *P < 0.001 compared with the model and scRNA groups; mRGCs, mouse retinal ganglion cells.

Since we previously observed decreased apoptosis upon *NgR* silencing, we analyzed the status of critical positive and negative regulators of apoptosis by analyzing the expression of Bcl-2, activated Bax, total Bax, pro-caspase3 and cleaved caspase3 in the NMDA-treated mRGCs by western blotting. We observed that all 4 groups of mRGCs demonstrated similar amounts of total Bax protein (*P* > 0.05). However, expression of the anti-apoptotic Bcl-2 and pro-caspase3 in the model and the nscRNA groups were significantly decreased compared to the blank and siNgR groups (*P* < 0.001) and the expression of pro-apoptotic activated Bax and cleaved caspase3 were significantly increased (*P* < 0.001; Figure 5). There was no significant difference in the expression of Bcl-2, activated Bax, pro-caspase3 and cleaved caspase3 between the model and nscRNA groups or the blank and siNgR groups (*P* > 0.05; Figure 5). These data suggested that pro-apoptotic signaling was significantly reduced when *NgR* was knocked down in the NMDA-treated mRGCs.

### NgR silencing decreased RhoA signaling in NMDA-treated murine retinal tissues

**Figure 6:**
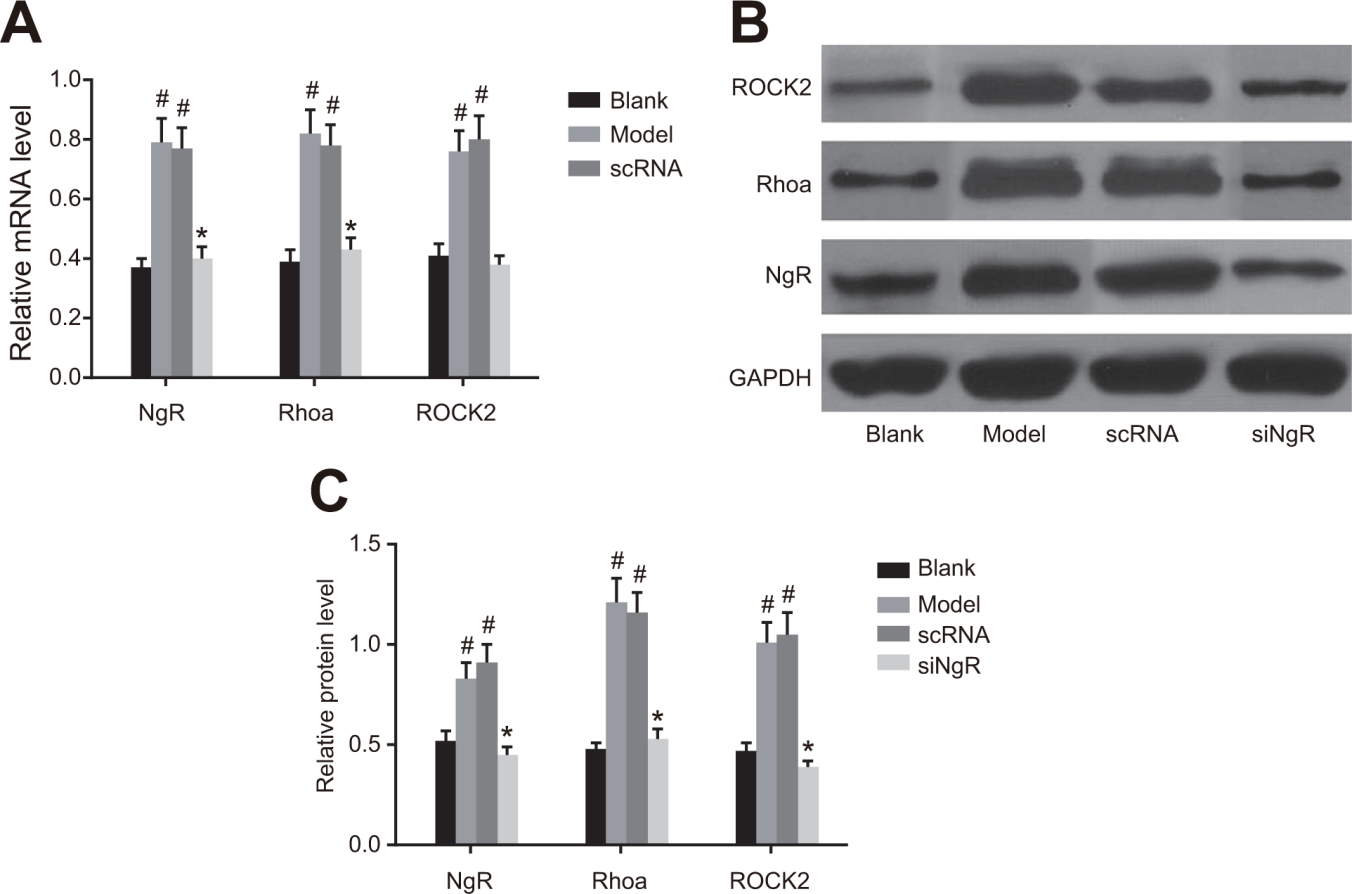
The effect of *NgR* silencing on RhoA signaling pathway in NMDA-treated murine retinal tissues (**A**) The expression of *NgR*, RhoA and ROCK2 mRNAs in the retinal tissues of the 4 groups of mice were analyzed by qRT-PCR. (**B**) The protein expression levels of *NgR*, RhoA and ROCK2 in retinal tissues of the 4 groups of mice were detected by western blotting. (**C**) The gray values of *NgR*, RhoA and ROCK2 protein bands in the retinal tissues of the 4 groups of mice is shown, ^#^*P* < 0.001 compared with the blank group; *P < 0.001 compared with the model and nscRNA groups; qRT-PCR, quantitative real-time polymerase chain reaction.

Next, we analyzed the effects of *NgR* silencing on RhoA signaling pathway in retinal tissues isolated from NMDA-treated mice by analyzing levels of *NgR*, RhoA and ROCK2 mRNAs and proteins by qRT-PCR and western blotting. We observed that the expression of *NgR*, RhoA and ROCK2 mRNA and proteins were significantly higher in the retinal tissues from the model and the nscRNA groups compared with those from the blank and the siNgR groups (*P* < 0.001; Figure 6). Comparatively, mRNA and protein levels of *NgR*, RhoA and ROCK2 were similar for the nscRNA and model groups as well as the blank and siNgR groups (*P* > 0.05; Figure 6). This further supported that *NgR* silencing results in decreased RhoA signaling in NMDA-treated murine retinas.

### NgR silencing decreased angiogenesis in NMDA-treated murine retinas

**Figure 7:**
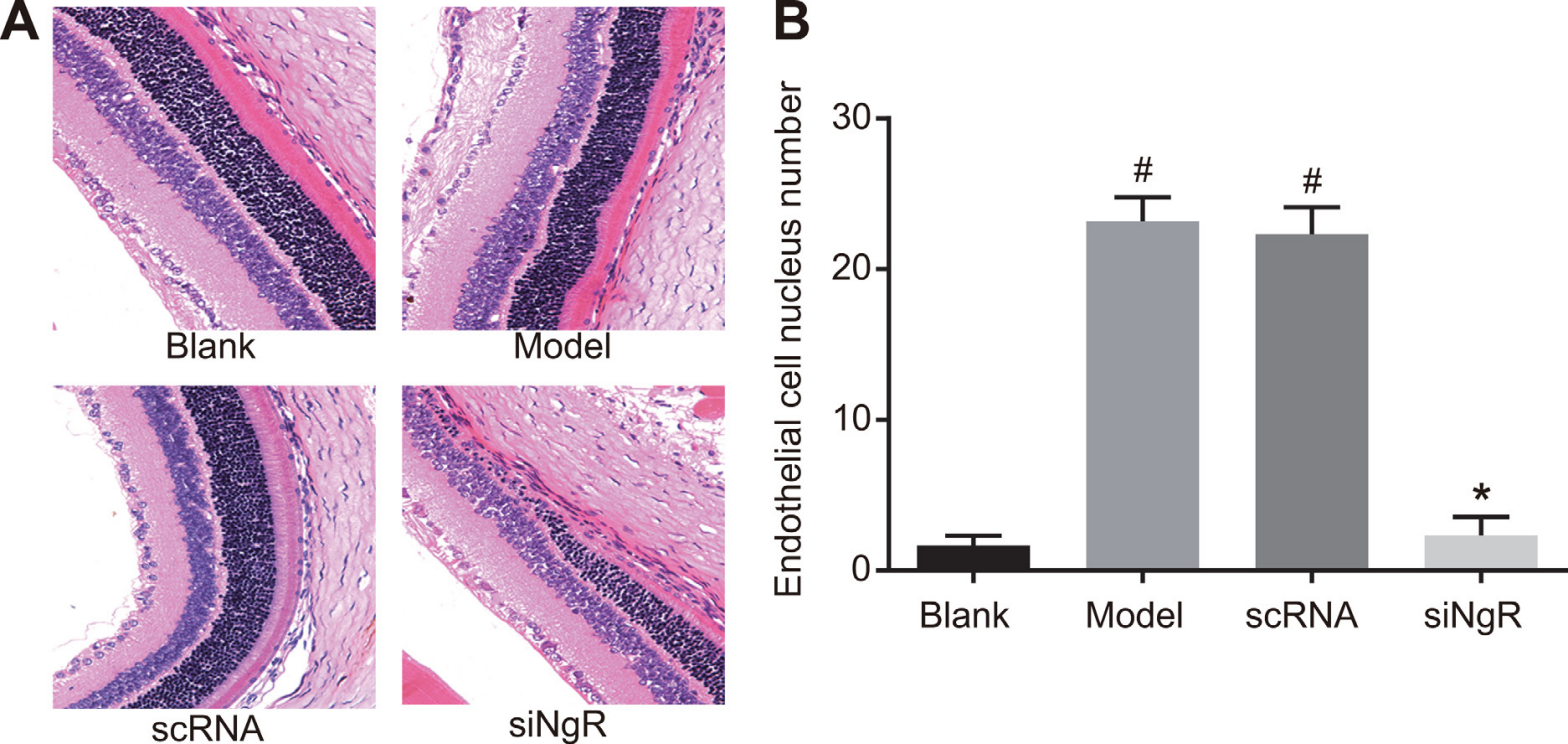
The effects of *NgR* gene silencing on angiogenesis in NMDA-treated murine retinal tissues (**A**) The arrangement of mRGCs in the H&E stained retinal tissue sections from the 4 groups of mice is shown. (**B**) The numbers of vascular endothelial cell nuclei that broke through the inner limiting membrane of the retina in the 4 groups were quantified (400X; black arrows represent endothelial cell nuclei). ^#^*P* < 0.001 compared with the blank group; *P < 0.001 compared with the model and nscRNA groups; H&E, hematoxylin and eosin.

Next, we analyzed the effects of *NgR* silencing on retinal angiogenesis by analyzing retinal histology of the 4 groups of mice. H&E staining showed regular, uniform and tight arrangement of mRGCs in the retinal tissues from blank and siNgR group mice (Figure 7A). In comparison, the mRGCs in the retinal tissues of model and the nscRNA group mice were significantly reduced and irregularly arranged (Figure 7A). This suggested that *NgR* silencing resulted in enhanced mRGC numbers and their structural integrity in the NMDA-treated murine retinas.

Further, we observed significantly higher number of vascular endothelial cell nuclei that broke through the inner limiting membrane of the retina in the retina of model and the nscRNA group of mice compared to the blank and the siNgR groups (*P* < 0.001; Figure 7B). There was no significant difference in the number of vascular endothelial cell nuclei that broke through the inner limiting membrane of the retina between the nscRNA and the model groups as well as the blank and siNgR groups (*P* > 0.05; Figure 7B). These data suggested that *NgR* silencing significantly reduced angiogenesis in the NMDA-treated murine retinas.

### NgR silencing increased mRGC density in NMDA-treated murine retinas

**Figure 8:**
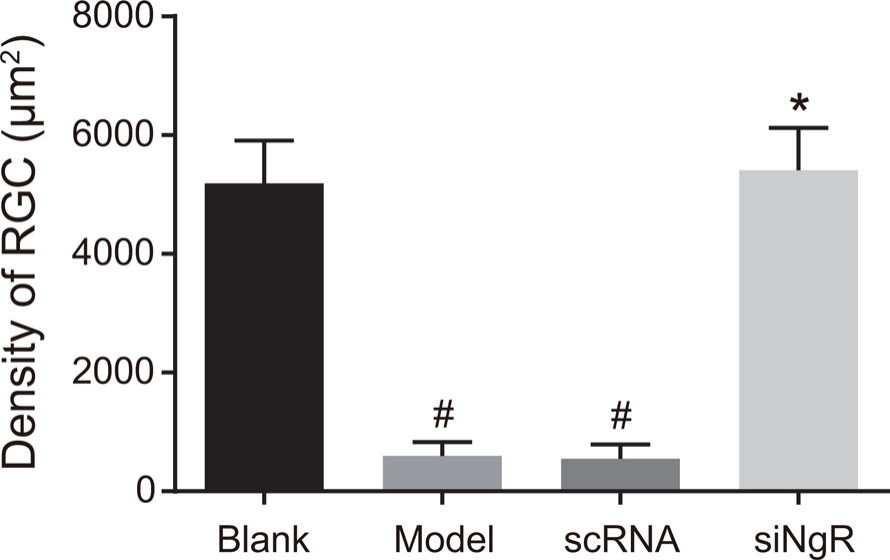
The effects of *NgR* gene silencing on mRGC cell density in NMDA-treated murine retinal tissues The number of mRGCs per unit area in the retinas of the 4 group of mice is shown. ^#^*P* < 0.001 compared with the blank group; *P < 0.001 compared with the model and nscRNA groups; mRGCs, mouse retinal ganglion cells.

Further, we analyzed the effects of *NgR* silencing on mRGC cell density in NMDA-treated murine retinas. We observed that mRGC density was significantly increased in the retinas of blank and siNgR groups compared with the model and the nscRNA groups (*P* < 0.001; Figure 8). Also, the density of mRGCs between the nscRNA and the model groups as well as the siNgR and the blank groups was similar (*P* > 0.05; Figure 8).

### Co-expression of Brn3a and NgR is maintained in NMDA-treated mRGCs

**Figure 9:**
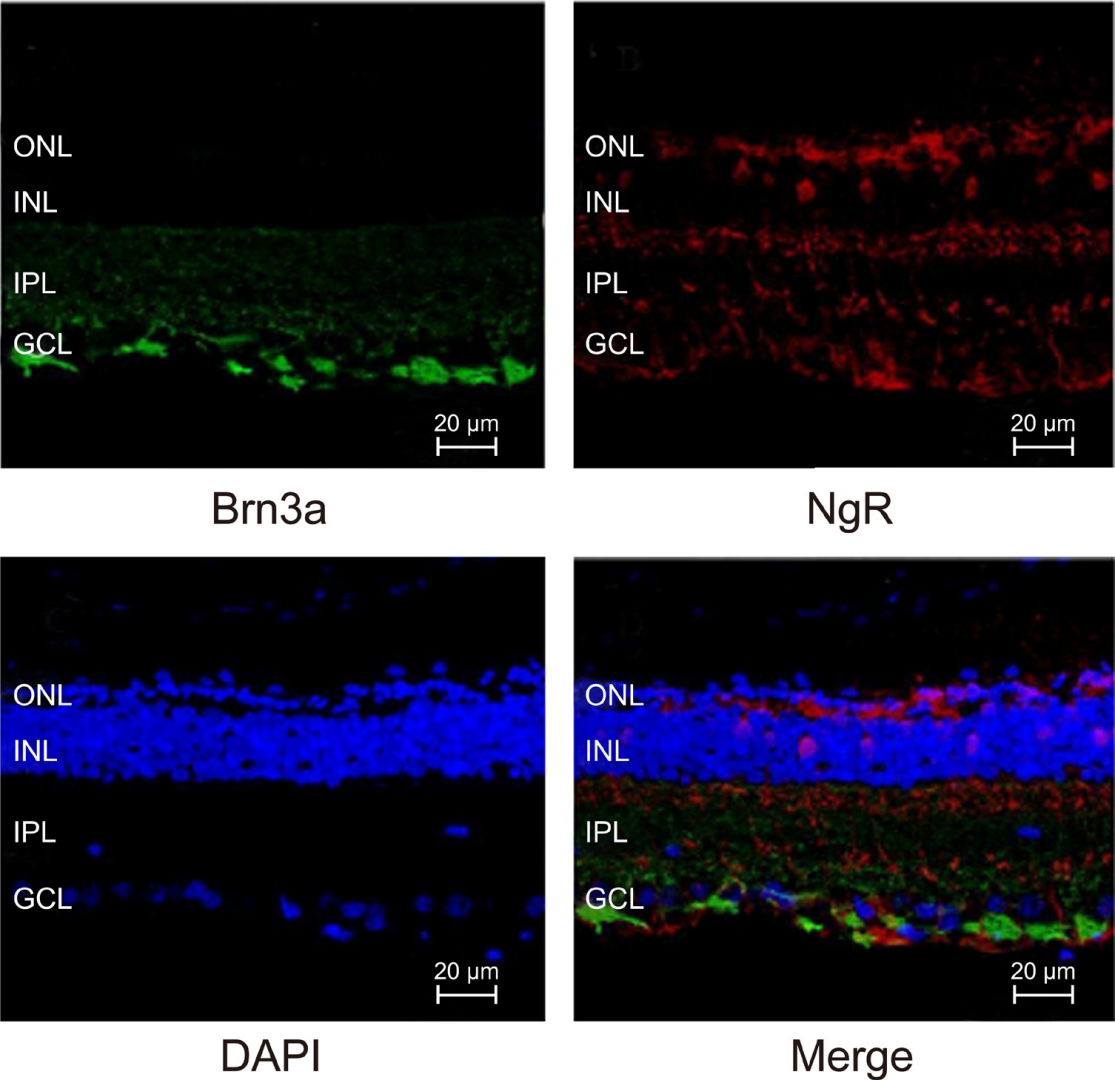
Immunofluorescence analysis of Brn3a and *NgR* expression in mRGCs from murine retinal tissues

We analyzed immunofluorescence stained sections of retinal tissues from the 4 groups of mice and found that both Brn3a (green fluorescence) and *NgR* (red fluorescence) proteins were co-localized in the mRGCs (Figure 9). Brn3a is a commonly used marker of the RGCs.

### NgR silencing decreased apoptosis in mRGCs from NMDA-treated murine retinas

**Figure 10:**
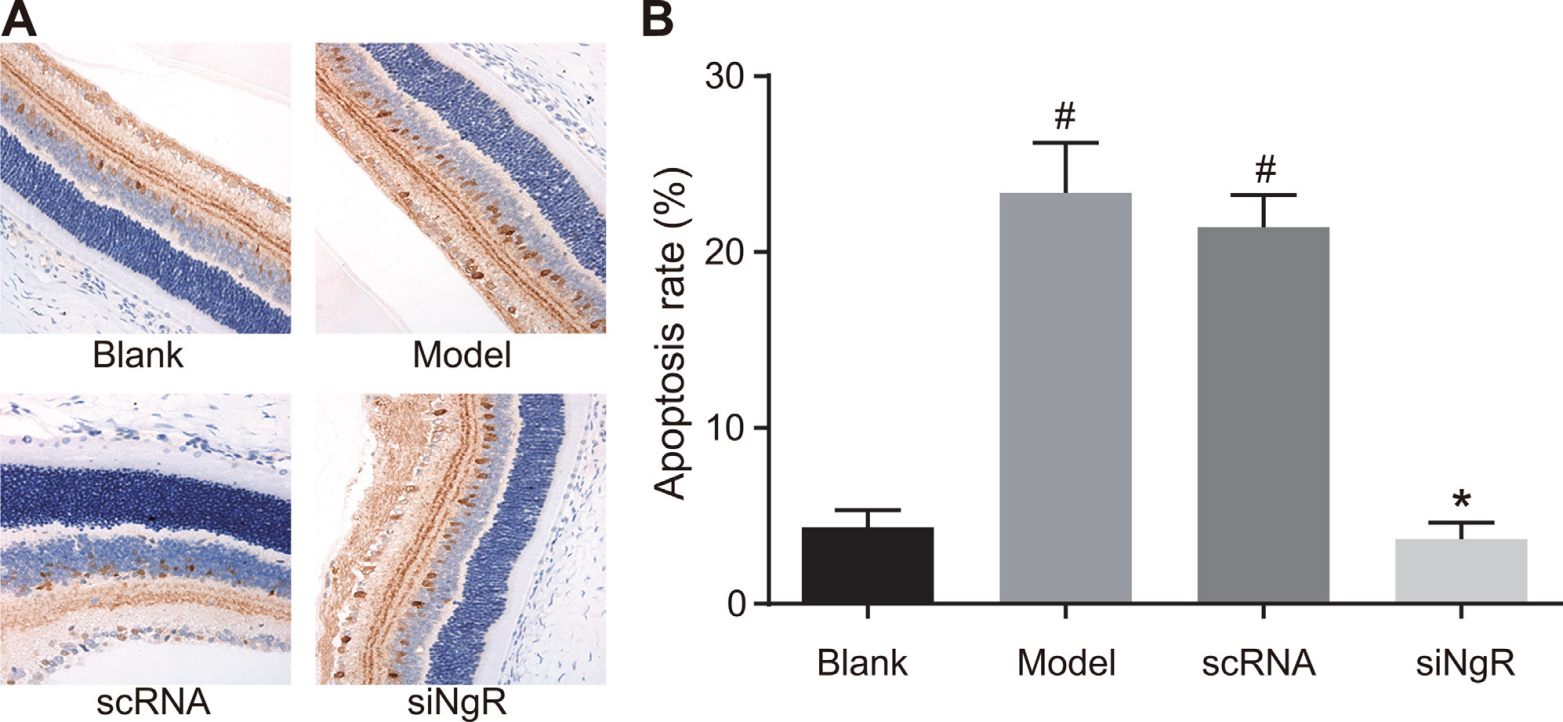
Effects of *NgR* silencing on mRGC apoptosis in NMDA-treated murine retinal tissues (**A**) TUNEL staining shows mRGC apoptosis in the retinal tissue sections of the 4 groups of mice. (**B**) Apoptotic rate was determined by quantifying the numbers of TUNEL- positive mRGCs in the murine retinal tissue sections from the 4 mice groups. ^#^*P* < 0.001 compared with the blank group; *P < 0.001 compared with the model and nscRNA groups; mRGCs, mouse retinal ganglion cells; TUNEL, terminal deoxynucleotidyl transferase dUTP nick-end labeling.

Finally, we analyzed the effects of *NgR* silencing on apoptosis of mRGCs in the retina of the 4 groups of mice by TUNEL staining. We observed that compared with the blank and siNgR groups, TUNEL positive cells were significantly increased in the retinas of the model and the nscRNA groups (*P* < 0.001; Figure 10). Also, the apoptotic rates of mRGCs in the retinas of nscRNA and model group mice as well as siNgR and the blank groups were comparable (*P* > 0.05). This suggested that *NgR* silencing reduced apoptosis in the mRGCs from NMDA-treated murine retinas.

## DISCUSSION

Acute energy reduction-induced death of RGCs is common in many ophthalmic diseases [17]. Apoptosis of RGCs in addition to axonal degeneration results in optic disc “cupping” and a gradual loss of vision [18]. The degree of nerve cell loss is closely related to the functional deficit [19]. Therefore, identifying factors and mechanisms that are involved in RGC apoptosis are important to identify therapeutic targets for optic nerve protection. Towards this goal, we investigated the effects of silencing *NgR* by lentivirus-mediated RNAi on the proliferation and apoptosis of mRGCs that are either treated with NMDA or from NMDA-treated murine retinas. We demonstrated that *NgR* silencing promoted proliferation and survival of mRGCs during NMDA induced optic nerve crush, thereby identifying *NgR* as a target for optic nerve protection in many ophthalmic diseases.

*NgR* is a common receptor for three myelin-associated inhibitor proteins namely, Nogo-A, myelin-associated glycoprotein (MAG) and oligodendrocyte myelin glycoprotein (OMGP), and is implicated in the failure of axonal regeneration in the adult mammalian central nervous system (CNS) [20]. Nogo-A is the most typical myelin-associated inhibitor that limits axon regeneration or functional recovery after CNS injury [21]. Anti-NogoA antibodies promoted growth of dorsal root ganglion neurites on CNS myelin, thereby providing proof of Nogo-A function [22]. RNAi mediated gene silencing is an effective, selective, and easily inducible method of suppressing expression of desired genes [23]. Therefore, we postulated that *NgR* silencing could promote the proliferation of mRGCs by accelerating their cell cycling and enhancing cell survival.

In this study, we observed that compared to the model and nscRNA groups, the expression of *NgR*, RhoA and ROCK2 in the siNgR groups were significantly decreased. The G protein RhoA and its downstream target ROCK2 are critical regulators of a variety of vasculature functions [24]. Nogo-A activates intracellular RhoA and ROCK resulting in collapse of the growth cone and repositioning axon guidance [25]. Thus, Nogo-A increases expression of RhoA and ROCK. Therefore, our study showed that silencing *NgR* decreased RhoA and ROCK2 mRNA and protein levels. Previously, it was shown that Nogo-A in combination with *NgR* activated the Rho-A GTPase, which together with ROCK inhibited neurite growth [26].

Our study also revealed that expression of anti-apoptotic Bcl-2 and pro-caspase3 increased and expression of pro-apoptotic activated Bax and cleaved caspase-3 decreased in the siNgR group compared with the model and nscRNA groups. This suggested that *NgR* silencing suppressed expression of activated apoptotic proteins, namely, activated Bax and cleaved caspase3 and enhanced expression of anti-apoptotic Bcl-2 and inactive pro-caspase3. The anti-apoptotic Bcl-2 is a therapeutic target of many human diseases [27, 28]. Caspase3 is a downstream executioner caspase that cleaves critical proteins of various cellular processes during apoptosis [29]. Bax is a pro-apoptotic protein of the Bcl-2 family that is stored in the cytosol in an inactive form and is activated by various stress and apoptotic stimuli [30]. It was previously demonstrated that inactivation of *NgR* suppressed apoptosis of RGCs [31]. Our data is in accordance with these findings.

Moreover, our study demonstrated that the density of mRGCs in the retinas of the siNgR group was significantly increased compared with the model and nscRNA groups. This suggested that *NgR* silencing could increase mRGC numbers and decrease retinal vascular proliferation, thus alleviating optic nerve injury. Previously, *NgR* expression was demonstrated in neurons as well as neural cells like neural stem cells, microglia and oligodendrocyte precursors [32]. Also, *NgR* is mainly expressed in the RGCs and activated *NgR* inhibits neural regeneration resulting in neuronal atrophy and apoptosis [14]. Supporting this, we demonstrate that lentivirus-mediated RNAi of *NgR* significantly decreases mRGC apoptosis and increases their density in the NMDA-treated murine retinas.

In conclusion, we demonstrate that *NgR* gene silencing results in increased proliferation and viability of the mRGCs in NMDA-treated retinas, thereby potentially alleviating optic nerve damage. Our findings suggest that inhibitors targeting *NgR* could therapeutically protect optic nerve function in many ophthalmic diseases and injury.

## MATERIALS AND METHODS

### Murine retinal ganglion cell culturing

The mRGCs were purchased from Shanghai Cell Bank of Chinese Academy of Sciences (Shanghai, China). The mRGCs were cultured in RPMI1640 media (Cat. No. A2494401, Gibco, Thermo Fisher, Waltham, MA, USA) containing 10% inactivated fetal bovine serum (FBS) (Cat. No. 10099141, Gibco, Thermo Fisher, Waltham, MA, USA) plus 100 units/ml penicillin and 100 mg/ml streptomycin (Cat. No. 15140122, Gibco, Thermo Fisher, Waltham, MA, USA) at 37°C and 5% CO_2_. The mRGCs were harvested with 0.25% trypsin when they reached 80% confluence.

### Construction of lentiviral vectors for NgR gene silencing

The *NgR* gene sequence (NM_022982.2; GenBank) was used to design the siRNA with the online RNAi design tool (https://rnaidesigner.thermofisher.com) provided by Invitrogen (Thermo Fisher, Waltham, MA, USA). The BLAST software (https://blast.ncbi.nlm.nih.gov/Blast.cgi) was used to confirm the unique siNgR sequence. Similarly, a non-specific control RNAi sequence (nscRNA) that did not match with any sequence in the human genome was also designed. The two siRNA sequences were synthesized by Sangon (Shanghai, China). The nscRNA sequence was 5ʹ-TGCCGTGCTAGATGGGGA-3ʹ and the siNgR sequence was 5ʹ-AATCTCACCATCCTGTGGCTG-3ʹ. The BamHI and HindIII restriction site sequences (D1010B, D1040B, TakaRa, Tokyo, Japan) were added to both ends of the designed oligonucleotide sequence, which was then inserted into a pSIREN-RetroQ-TetH lentiviral vector (TakaRa, Tokyo, Japan). The recombinant plasmids were amplified by Escherichia coli DH5α and cultured overnight in LB medium containing ampicillin. The amplified plasmids were extracted according to the instructions using the plasmid mini preparation kit (D0005, Beyotime, Shanghai, China) and verified by sequencing (Invitrogen, Thermo Fisher, Waltham, MA, USA).

### *In vitro* RNAi of NgR in mRGCs

The experimental design included four groups as follows: (1) Blank group that was cultured in normal growth conditions; (2) Model group to determine the effect of NMDA treatment (3) The nscRNA group that was transfected with the nscRNA lentiviral vectors; and (4) The siNgR group that was transfected with the nscRNA lentiviral vectors. For the model, nscRNA and siNgR groups, 100 μM N-methyl-D-aspartate (NMDA, No.G0541, Sigma, St. Louis, MO, America) and 10 μM glycine was added for 30 min and then washed with phosphate buffered saline (PBS) thrice before being cultured in the original growth medium.

Transduction of lentiviral vectors were performed according to the instructions of the Fugene transduction kit (E2311, Promega, Madison, WI, USA). The mRGCs were cultured in a 6-well plate for 24 h before transduction. Fresh 2 mL RPMI1640 medium was added 2 h before the transduction. The transduction reagent Fugene (8 μL) was mixed with 3.2 μg plasmids for 20 min at room temperature. After aspirating the RPMI1640 culture medium, 200 μL opti-MEM medium was added into each well, followed by the addition of 800 μL of the Fugene/plasmid mixture. Then, the transfected cells were incubated at 37°C for 5 h followed addition of 2mL RPMI1640 medium into each well and incubated at 37°C.

### Analysis of mRGC proliferation by CCK 8 assay

The mRGCs were seeded at a density of 10^3^ cells in 200 μL medium/well in a 96-well plate. There were 5 duplicated wells for each of the blank, model, nscRNA and siNgR groups. From 0–3 days, 20 μL CCK-8 reagent was added into one well of each of the 4 groups and the optical density (OD) was measured at 490 nm using a microplate reader after 4 h and plotted against time. On the 3rd day, the number of cells in the four groups were also counted under a microscope and photographed. The experiments were repeated thrice for each group.

### Analysis of mRGC cell cycle by flow cytometry

The four experimental groups of mRGCs (blank, model, nscRNA and siNgR) were cultured for 3 days and after washing once with PBS, they were fixed in PBS containing 75% ethanol and 0.5 mM EDTA at 4°C for 1 h. Then, the cells were centrifuged at 2000 rpm for 5 min, washed with PBS and resuspended in 500 μL PBS containing 0.1% Triton X-100 and 50 μg/mL RNAase. The cells were then quickly stained by adding 90 μL of 0.5 mg/mL propidium iodide (PI) staining solution and after gently mixing with a pipette, the cells were incubated at room temperature in the dark for 30 min. Then, the cells were filtered by a nylon membrane and analyzed by an EPICS XL-4 flow cytometer (Beckman Coulter, Brea, CA, USA). Experiments were repeated thrice for each group.

### Analysis of mRGC apoptosis by flow cytometry

The four experimental groups of murine RGCs (blank, model, nscRNA and siNgR) were cultured for 3 days, washed twice with PBS, digested with 0.25% trypsin and centrifuged at 1000 rpm for 10 min. The cells were collected, washed thrice with PBS and the cell concentration was adjusted to 5 × 10^5^ cells/mL. Then, 5 μL AnnexinV-FITC was added to 100 μL cells and incubated at room temperature for 10 min in the dark followed by centrifugation at 1000 rpm for 5 min. Then, 10 μL PI staining solution was added to the AnnexinV-FITC stained cells and analyzed by flow cytometry to determine the percentage of AnnexinV^+^/PI^+^ cells that represent the apoptotic cells. Experiments were repeated thrice for each group.

### *In vivo* mouse model to study effect of NgR gene silencing on NMDA-treated retinal mRGCs

Mouse experiments were designed with the consent of the Animal Ethics Committee from our institution and all the studies were conducted strictly in accordance with the National Institutes of Health (NIH) guidelines for animal care and application. Specific pathogen free (SPF) grade 8-week old male C57BL/6 mice were provided by Shanghai Nanfang Experimental Animal Center. Mice were randomly divided into 4 groups (blank, model, nscRNA and siNgR groups) with 12 mice per group. The mice had free 24 h access to food and water and were housed in a 12 h day/night cycle. All mice were allowed to adapt for 7 days prior to the experiment. In the model, nscRNA and siNgR groups, 100 μM NMDA was injected through the sclera, 3 mm from the outside edge of superior temporal limbus. After the needle tip reached the posterior portion of the vitreous body, the liquid was injected slowly. The mice were injected similarly for 3 days. Lincomycin eye drops were given to all mice to prevent infections and any blind mice were removed. In the blank group, the same volume of saline was injected instead of NMDA. Then, the mice in the nscRNA and siNgR groups were injected with 10 μL (8 μL Fugene plus 3.2 μg plasmids) liposome-coated nscRNA or siNgR lentiviral vectors, respectively. In the blank and model groups, mice were injected with 10 μL of liposomes and water (4: 1) mixture. The mixture was injected four times, once every 10 days. The day after the last injection, 12 mice from each group were sacrificed and their right eyeballs were dissected. After removing the cornea, lens and vitreous tissues, the remaining eye cup was wrapped in tin foil and placed in liquid nitrogen for future use.

### Quantitative real-time polymerase chain reaction (qRT-PCR)

Total RNA was extracted from the 4 groups of mRGCs (blank, model, nscRNA and siNgR groups) that were cultured for 3 days as well as the retinal tissues that were harvested from the four groups of mice and stored in liquid nitrogen according to instructions from the RNA extraction kit (Z3100, Promega, Madison, WI, USA). For RNA quantification, ratio of OD values at 260 nm and 280 nm (A_260_/A_280_) were determined for all samples by an ultraviolet spectrophotometer. The RNA samples were stored at –80°C for future use.

For qRT-PCR analysis of *NgR*, primers were designed based on the gene sequence in the Genebank database using the Primer6.0 primer design software and synthesized by Sangon (Shanghai, China). The primer sequences were as follows: GAPDH, GCCAGCC TCGTCTCATAGACA (forward) and TGGTAACCAGG CGTCCGATA (reverse); *NgR*, AATGAGCCCAAGGT CACAA (forward) and CCATGCAGAAAGAGATGCGT (reverse); RhoA, GCAGGTAGAGTTGGCTTTATGG (forward) and CTTGTGTGCTCATCATT (reverse); ROCK2, GAACCTACTCCTGGAAGCCG (forward) and TGCTTCAGCAGCTCATTCAGTTT (reverse) (Table 2). Reverse transcription was performed according to the protocol provided by a RNA reverse transcription kit (A3500, Promega, Madison, WI, USA).For quantitative PCR, the protocol included pre-denaturation at 95°C for 15 min, followed by 40 cycles of the denaturation at 95°C for 10 s, annealing at 60°C for 30 s and extension at 72°C for 30 s. The PCR reaction mixture included 12.5 μL SYBR Green Mix, 1 μL forward primer, 1 μL reverse primer, 2 μL cDNA template and 8.5 μL ddH_2_O (Cat. No. 4367659, Thermo Fisher, Waltham, MA, USA). GAPDH was used as the internal reference. The dissolution curve was used to determine the reliability of the PCR results. The cycle threshold (CT) values were used to determine the relative expression of the target gene compared to the control GAPDH using the 2^-ΔΔCt^ method. The experiments were repeated thrice for each group with 12 mice in each group.

**Table 2:** The primer sequences for qRT-PCR

Gene	Forward (5′–3′)	Reverse (5′–3′)
GAPDH	GCCAGCCTCGTCTCATAGACA	TGGTAACCAGGCGTCCGATA
NgR	AATGAGCCCAAGGTCACAA	CCATGCAGAAAGAGATGCGT
Rhoa	GCAGGTAGAGTTGGCTTTATGG	CTTGTGTGCTCATCATT
ROCK2	GAACCTACTCCTGGAAGCCG	TGCTTCAGCAGCTCATTCAGTTT

Note: qRT-PCR, quantitative real-time polymerase chain reaction.

### Western blotting

After mRGCs were cultured for 3 days, the proteins of mRGCs in the four experimental groups were extracted. Also, the murine retinal tissues from the 4 experimental groups that were stored in liquid nitrogen were homogenized and soluble protein lysates were prepared. The protein concentrations in cell and tissue lysates were determined according to the BCA kit protocol (Cat. No. AR0145, Boster Biological Engineering Co., Ltd., Wuhan, China). The extracted proteins (30 μg) were denatured at 95°C for 5 min in loading buffer. Then, the protein samples were separated on a 10% SDS-PAGE at 80–120 V followed by wet transfer at 100 mV for 45–70 min onto PVDF membrane. After blocking the membrane for 1 h with 5% BSA at room temperature, the membrane was incubated overnight at 4°C with primary antibodies for *NgR* (1: 1000, ab184556, Abcam, Cambridge, UK), RhoA (1: 5000, ab187027, Abcam, Cambridge, UK), ROCK2 (1: 1000, ab183636, Abcam, Cambridge, UK), Bcl-2 (1: 1000, ab32124, Abcam, Cambridge, UK), total Bax (1: 1000, ab32503, Abcam, USA), activated Bax (1:1000, ALX-804-224-C100, Enzo, Farmingdale, NY, USA), cleaved caspase3 (1:1000, 9661S, CST, San Antonio, TX, USA) and pro-caspase3 (1:1000, ab32150, Abcam, USA). Subsequently, the membrane was rinsed thrice with TBST for 5 min each. Then, the corresponding secondary antibodies were added and incubated at room temperature for 1 h. The membrane was again washed thrice for 5 min each. GAPDH was used as an internal reference (1:5000, KC-5G5, Kangcheng Biological Engineering Co., Ltd., Shanghai, China). Experiments were repeated thrice. A Bio-rad Gel Dol EZ imager (Bio-rad, Hercules, CA, USA) was used to develop the images. The target protein band was quantified using the Image J software.

### Hematoxylin and eosin (HE) staining of murine retinal tissue sections

After conventional eyeball sectioning, 1 slice was selected for every 6 slices (6 μm thick; 10 slices per eyeball). The slices were stained with hematoxylin and eosin (H&E). After H&E staining, the slices were washed followed by gradient dehydration by ethanol. Then, the slices were made transparent with xylene, mounted and imaged under a light microscope. In each slice, 10 fields were chosen at high magnification (400X). The average numbers of vascular endothelial cell nuclei that broke through the inner limiting membrane of the retinas were determined for each eyeball. While counting the numbers of vascular endothelial cell nuclei, endothelial cells that were closely related to the inner limiting membrane were counted and the endothelial cells in the vitreous cavity without connection to the internal limiting membrane were excluded. Analysis was performed for eyeballs from 12 mice in all 4 groups.

### Retinal slice preparations and counting of mRGCs

On the second day after the last injection, the retinas from mice in all four groups were fixed in 4% paraformaldehyde solution. The eyeballs were collected and the cornea, iris, lens and vitreous body were removed. The remaining eye tissues were then placed in PBS containing 30% sucrose and incubated for 12 h. Saggital sections of the frozen retina were then prepared. Subsequently, the retinal slices were stained with hematoxylin staining, followed by color separation, washing and gradient dehydration by ethanol. Then, the slices were treated with xylene, mounted and imaged under a microscope. For each slice, 5 fields were randomly selected and the number of ganglion cells was counted. The average numbers of mRGCs in each group were determined and the density of mRGCs per unit area of each field was calculated for all mice in the 4 groups.

### Immunofluorescence analysis of Brn3a and NgR in mRGCs of murine retinal tissue sections

On the second day after the last injection, the eyeballs from all mice from the 4 groups were dissected. The cornea, iris, lens and vitreous body were removed and the remaining eye tissues were fixed with 4% paraformaldehyde solution for 40 min. Then, the sclera and choroid were carefully peeled off under a microscope. An incision was made along the two-thirds of the line connecting the serrated edge and the center of the optic disc. The tissues were then stretched on glass slides (with the vitreous cavity facing up), and samples were blocked with 5% BSA for 24 h at 4°C. Further, the samples were incubated with diluted primary antibodies against Brn3a (1: 1000, ab35376, Abcam, Cambridge, UK) and *NgR* (1: 1000, ab184556, Abcam, Cambridge, UK) at 4°C overnight. Then, the slides were washed and subsequently incubated with the Cy3-labeled secondary antibody in the dark for 1 h at 4°C. After washing, the FITC-labeled secondary antibody was added and incubated in the dark for 1 h at 4°C. Then, the slides were incubated with DAPI (1: 500, D9542, Sigma, St. Louis, MO, USA) for 10 min and washed thrice with TBST for 5 min each. After stretching the tissues again, the slides were mounted and photographed with a fluorescence microscope (OLYMPUS, Tokyo, Japan) in the dark.

### Terminal deoxynucleotidyl transferase dUTP nick-end labeling (TUNEL) staining

The retinal sections were digested by 0.1% trypsin for 3 min. After rinsing twice with PBS for 5 mins each, the sections were incubated with 0.3% H_2_O_2_ methanol solution at room temperature for 30 min. Then, they were incubated with 0.1% TritonX-100 for 2 min followed by incubation with the TUNEL reaction mixture in a wet box for 1 h at 37°C. Then, the converter-POD was added onto the slides and incubated in a wet box for 30 min at 37°C. The sections were then developed with DAB at room temperature for 5 min. Subsequently, they were stained with hematoxylin, washed with distilled water, dehydrated, made transparent, mounted and photographed under a microscope. The positive cells showed brown particles in the nuclei. Randomly, 5 fields were selected in each slide and the numbers of positive cells were counted (200X). The apoptosis rate was calculated as the percentage of TUNEL-positive cells in the 4 groups.

### Statistical analysis

The experimental data were statistically analyzed by the SPSS21.0 statistical software. The data were expressed as mean ± standard deviation (S.D). The significance of differences between two groups of data was analyzed using the *t* test. The comparison among multiple groups was performed with one-way analysis of variance (ANOVA). The enumeration data were expressed as cases, frequency or percentage using χ^2^ test. *P* < 0.05 was considered statistically significant.
